# Applications of Radio Frequency Heating in Food Processing

**DOI:** 10.3390/foods12061133

**Published:** 2023-03-08

**Authors:** Shaojin Wang, Rui Li

**Affiliations:** 1 College of Mechanical and Electronic Engineering, Northwest A&F University, Xianyang 712100, China; 2 Department of Biological Systems Engineering, Washington State University, Pullman, WA 99164-6120, USA

Considering safety concerns regarding postharvest agricultural products or foods, environmental pollution caused by chemical fumigations, and increased international regulations to limit the use of fumigants, it is an extremely urgent task to develop novel and environmentally friendly physical alternatives to the postharvest control of insect pests and pathogens. Radio-frequency (RF) treatment has been identified as a novel physical heating method, providing fast and volumetric heating. The purpose of this Special Issue is to focus on the recent developments and applications of RF heating in food and agricultural product processing, such as disinfestations, drying, pasteurization, sterilization, roasting, temping and thawing. This Special Issue aims to present the most significant methods, research strategies and protocols used in the development of environmentally friendly food processing based on RF energy.

This Special Issue collates 13 papers related to RF processing technology. The thermal and dielectric properties of agricultural products [1] are important for understanding RF heating principles and necessary for establishing a computer simulation model to improve RF heating uniformity in foods [2,3]. RF drying has been widely used to replace conventional dehydration methods for improving product quality and storage stability [4,5,6]. Based on thermal inactivation kinetics, RF energy has been applied to control pests and pathogens [7,8,9] in foods and agricultural products, shortening treatment time but maintaining product quality [10]. RF heating could also be used in the food industry to roast nuts since RF-roasted almonds were found to have a better flavor, texture, and overall preferability compared to commercial almonds [11]. Finally, RF heating has been successfully used for the tempering and thawing the frozen foods by reducing the drip and micronutrient losses [12,13].

In this Special Issue, we hope to establish a sound basis for the further development of thermal methods for the RF control of pests and pathogens, as well as the drying, roasting and thawing of foods and harvested agricultural commodities. This Special Issue will form an important resource for readers who are interested in the knowledge, methods and strategies used in the development of environmentally friendly RF processes. This Special Issue may also be suitable for researchers who work on heating technologies relevant to RF all over the world.

The editorial team would like to express its gratitude to the contributors for sharing their novel ideas, new knowledge, and innovative findings. Each paper has been handled by a qualified editorial team and reviewed by two international experts in RF fields. Therefore, we thank those reviewers for helping us maintain a high standard in the Special Issue. We hope to maintain a strong understanding, collaboration, and friendship with our colleagues across disciplinary, institutional, and country border lines.

## Data Availability

Not applicable.
